# Impact of a program of training on the performance of a track and trigger system and outcome of ICU admissions

**DOI:** 10.1186/cc9892

**Published:** 2011-03-11

**Authors:** M Waraich, J Zwaal, M Johnson

**Affiliations:** 1 Kingston Hospital, Kingston Upon Thames, UK

## Introduction

The introduction of track and trigger systems for hospitalised patients has been associated with improvement in outcome through earlier identification of sick patients [1]. We set out to improve the performance of our track and trigger system through a program of intense training of frontline medical and nursing staff with the aim to improve outcome of patients admitted to ICU.

## Methods

A retrospective chart survey of all ICU admissions from the ward 3 months before and after conclusion of a program of training was carried out. Out of a total of 64 charts, four were incomplete and three concerned planned postoperative ICU admissions and were therefore omitted from analysis. Training emphasized triggering of the pathway if two or more physiological parameters were outside the normal range: respiratory rate <10 or ≥25, SaO_2 _< 90%, systolic blood pressure <90, heart rate <50 or ≥110 and/or neurological response to painful stimulus only. The pathway could also be triggered by clinical concern about the patient. Triggering progressed through involvement of junior medical and nursing staff at step 1, intermediate level at stage 2 and senior medical and ICU staff at step 3. Outcome parameters: compliance with pathway steps and mortality of ICU admissions. Differences between proportions were tested according to the method described by Armitage and colleagues [2] with *P *< 0.05 taken as significant.

## Results

Significant improvement was found in triggering at steps 1 and 3 with a reduction in noncompliance at step 1 from 51.7% to 28.1% (*P *= 0.044) and at step 3 from 31% to 9% (*P *= 0.018). The pathway compliance overall showed a nonsignificant improvement from 17.2% to 33.3%. Pathway noncompliance showed a trend towards occurrence out of hours (70% vs. 60.5%). Pathway sensitivity was unchanged (69% before vs. 61.4% after). There was no difference in ICU mortality pre and post training (41.4% vs. 47.4%, *P *= 0.6). Neither was there a difference in ICU mortality between pathway followers and nonfollowers (57.9% vs. 42.1%, *P *= 0.95).

## Conclusions

Improved performance of the track and trigger system did not lead to improved outcome in patients admitted to the ICU.
